# Blaze a New Trail: Plant Virus Xylem Exploitation

**DOI:** 10.3390/ijms23158375

**Published:** 2022-07-29

**Authors:** Yong-Duo Sun, Arianna Spellman-Kruse, Svetlana Y. Folimonova

**Affiliations:** 1 Department of Plant Pathology, University of Florida, Gainesville, FL 32611, USA; arianna.spellman@ufl.edu; 2 Plant Molecular and Cellular Biology Program, University of Florida, Gainesville, FL 32611, USA

**Keywords:** plant virus, vascular loading, xylem movement, virus–xylem interactions

## Abstract

Viruses are trailblazers in hijacking host systems for their own needs. Plant viruses have been shown to exploit alternative avenues of translocation within a host, including a challenging route through the xylem, to expand their niche and establish systemic spread, despite apparent host-imposed obstacles. Recent findings indicate that plant viruses from many families could successfully hack xylem cells in a broad range of plant hosts, including herbaceous and perennial woody plants. Similar to virus-related structures present in the phloem, virus particles and membrane-containing viral replication complexes are often observed in the xylem. Except for a few single-stranded DNA viruses in the family *Geminiviridae* and a negative-sense single-stranded RNA rhabdovirus, *Lettuce necrotic yellows virus*, the majority of the viruses that were detected in the xylem belong to the group of positive-sense RNA viruses. The diversity of the genome organization and virion morphology of those viruses indicates that xylem exploitation appears to be a widely adapted strategy for plant viruses. This review outlines the examples of the xylem-associated viruses and discusses factors that regulate virus inhabitation of the xylem as well as possible strategies of virus introduction into the xylem. In some cases, plant disease symptoms have been shown to be closely related to virus colonization of the xylem. Inhibiting viral xylem invasion could raise potential attractive approaches to manage virus diseases. Therefore, the identification of the host genes mediating virus interaction with the plant xylem tissue and understanding the underlying mechanisms call for more attention.

## 1. Introduction

In plants, the xylem tissue functions to transport water and minerals unidirectionally, from roots towards the aboveground parts [1,2]. At the same time, the phloem serves as a highway for the bidirectional distribution of sugars, nutrients, and hormones, in a source-to-sink manner. Apparently, the microaerophilic environment of the phloem is thought to provide a suitable niche for plant-infecting viruses. In the past decades, considerable knowledge of the phloem structure and its role in virus colonization has been gained. It is widely accepted that plant viruses utilize the phloem as the main route for the long-distance spread within a host, along with photoassimilate translocation, and, thus, the virus–phloem interplay has been a hot area in plant virus research [3,4,5]. However, virus–xylem interactions have been largely overlooked. On the one hand, functional xylem cells are mainly dead cells, which lack the cellular machinery that the virus needs for the synthesis of its proteins, replication, and assembly of new particles. On the other hand, the movement of a virus from an adjacent cell containing a protoplast to the apoplastic compartment of the xylem requires passing across an intact plasma membrane and a thickened secondary cell wall. With that, the xylem seems to be a challenging territory for the plant viruses and an unlikely area to serve for virus occupation. Interestingly, a growing number of studies, especially those in more recent years, point to the importance of the relationship between the vascular xylem in the plant host–virus interactions and disease production [6,7,8]. Viruses do find ways out to blaze trails in the xylem, as they constantly evolve and adapt to environmental challenges. Some studies even suggest that, for specific viruses, active virus accumulation takes place in immature xylem-associated cells, rather than in phloem-associated cells. For instance, virions of *Lettuce necrotic yellows virus* (LNYV) were not observed in the thin sections of the phloem cells of tobacco (*Nicotiana glutinosa* L.). Instead, the virus particles were successfully visualized in juvenile xylem cells [9]. With evidence generated from these studies, it seems that at least some plant viruses can manipulate xylem cells to replicate and spread to distal plant parts.

Research on the virus-xylem interplay can be traced back to the 1930s when Johnson revealed the presence of *Tobacco mosaic virus* (TMV) in the xylem guttation from tomato (*Solanum lycopersicum* L.) [10]. Since guttation fluid is the exudation of xylem sap, finding virus particles in this exudate demonstrated that infectious TMV could spread via the xylem conduit. Later, the presence of several plant viruses was observed in the xylem of many herbaceous plant hosts. Virions of *Beet necrotic yellow vein virus* (BNYVV) were frequently found in the undifferentiated and mature xylem vessel elements and xylem parenchyma of sugar beet (*Beta vulgaris* L.) [11]. The infectious virions of *Tomato mosaic virus* (ToMV) and *Pepper mild mottle virus* (PMMV) were recovered and visualized from the guttation fluid of tomato (*Lycopersicon esculentum* Mill.) and green pepper (*Capsicum annuum* L.), respectively [12]. With such evidence on hand, French and colleagues tested the potential capability of a diverse group of viruses to move in the xylem sap. Strikingly, the respective results showed that plant viruses from at least ten genera could employ the xylem tissue as a conduit for their transportation [13]. Compared to those in the herbaceous hosts, studies on virus–xylem interactions in perennial woody plants are limited. The reason could be due largely to the fact that fewer viruses have been reported to infect the xylem of a tree. Some time ago, *Blueberry shoestring virus* (BSV) was shown to achieve the long-distance movement through the xylem, in addition to the phloem of a low woody shrub, highbush blueberry (*Vaccinium corymbosum* L.) [14]. Furthermore, a recent study on *Citrus tristeza virus* (CTV) reignited an interest in exploring virus–xylem interactions in perennial woody plants by demonstrating that CTV can invade and further develop a ‘clustered oasis’ of infected cells in the xylem tissue of *Citrus macrophylla* Wester [7]. It is possible that hijacking the xylem cells could be a strategy adapted by plant viruses to promote infection in a broad range of plant host. 

The healthy and functional xylem of a plant is important to its vigorous growth. The invasion of certain vascular pathogens was shown to damage the xylem function and trigger disease syndromes. For instance, a xylem-residing *Verticillium dahliae* Klebahn, a filamentous fungus, could secret a toxic protein named xylanase 4 to degrade the xylem’s cell walls and induce necrosis. The destruction of the xylem vessels results in wilt syndrome in the infected cotton plants [15]. In contrast, the connection between the virus invasion of the xylem and disease development has not been clearly demonstrated, largely because the vascular xylem is not the main or the sole tissue that the virus colonizes. Other tissues, such as the epidermis, mesophyll, cortex, and phloem, provide more suitable niches for plant viruses, and the phenotypes induced by the virus in those tissues are usually more easily noticeable. For instance, while *Brome mosaic virus* (BMV) is able to invade xylem cells in oat (*Arena sativa* L.) and barley (*Hordeum vulgare* L.), leaf mosaic symptoms are attributed to the virus accumulation in the juvenile phloem and mesophyll cells [16]. In contrast, there is an exception where CTV triggers an obvious phenotype in the vascular tissues of several different citrus varieties, including the xylem. Remarkably, the invasion of CTV into the immature xylem treachery elements and ray parenchyma cells in the citrus plants was shown to account for the development of one of the major disease syndromes induced by this virus. Such invasion interrupts the differentiation of the xylem and phloem cells in the vasculature and eventually triggers the stem pitting disease, which is accompanied by the loss of tree vigor and production of unmarketable fruit. The manifestation of this syndrome in the citrus trees may emerge months or years after CTV inoculation [7,17]. Such phenotype in the CTV-colonized citrus stems and branches could be contingent upon the continuous growth of the xylem rings and the virus xylem invasion. Consequently, the severity of disease is enhanced with tree growth. It is possible that some xylem-invading viruses share similar mechanisms to trigger diseases in other hosts. 

There is no doubt that virus invasion in the xylem has critical outcomes for plant health and disease production in crops. Nonetheless, our understanding of the virus–xylem interactions is still in its infancy. Thus, it would be important to understand the connection of virus xylem invasion and disease manifestation.

## 2. Examples of Xylem-Invading Viruses

To date, at least 39 plant viruses have been reportedly found in the xylem-associated cells of many different hosts, including herbaceous and perennial woody plants (Table 1). Interestingly, the majority of xylem-invading viruses (35/39) studied to date belong to the group of positive-sense single-stranded RNA (+ssRNA) viruses in the following families: *Alphaflexiviridae*, *Benyviridae*, *Bromoviridae*, *Closteroviridae*, *Potyviridae*, *Secoviridae*, *Sobemoviridae*, *Tombusviridae*, and *Virgaviridae* (Table 1) [6,7,8,10,11,12,13,14,16,18,19,20,21,22,23,24,25,26,27,28,29,30,31,32,33,34,35,36]. Other viruses are three single-stranded DNA (ssDNA) viruses in the family of *Geminiviridae* [37] and a negative-sense RNA virus in the family *Rhabdoviridae* (Table 1) [9]. With such a broad range of virus families, the morphology of xylem-invading viruses is quite diverse. Virions produced by those viruses could be long flexuous or short rigid rod-like particles or have icosahedral, twinned-icosahedral, or bullet-like shape and range from approximately 10 nm to over 2000 nm (Table 2). The diversity of these viruses suggests that the xylem exploitation appears to be a widely adapted strategy for plant viruses, which might be an evolutionary consequence of the virus niche expansion.

## 3. Techniques for Studying Virus Invasion into the Xylem

The fact that the xylem tissue is located deep inside a plant presents certain challenges for studying virus–xylem interactions. Thus, the limitation of available techniques has hindered the development of research in this area [14]. Early experiments used approaches based on plant physiology to analyze plant guttation fluid, which originates from the exudation of xylem sap, and assess the virus presence in the host xylem. Virus detection and diagnosis in the guttation has been achieved using reverse transcription-polymerase chain reaction, enzyme-linked immunosorbent assay, and transmission electron microscopy (TEM). By integrating these methods, several plant viruses have been found in the guttation fluid of a variety of plant species, such as TMV in tomato, PMMV in green pepper, ToMV in tomato, LNYV in tobacco, *Cucumber necrosis virus* (CNV) in cucumber (*Cucumis surivus* L.), and BMV in wheat (*Triticum aestivum* L.) and barley [9,10,12,13,20]. However, the limitation of analyzing guttation fluid is that it could be easily contaminated during sampling since the xylem tissue is closely attached to the phloem in the vasculature. To avoid this contamination, additional approaches to separate the phloem and xylem tissues are employed. For instance, girdling a stem section results in the removal of the bark around the circumference of a section in the main stem, or treating a stem with a hot steam allows to deplete the phloem tissue while maintaining the integrity of the functional xylem. The mechanical stem-girdling experiment could be easily conducted with perennial woody plants, considering the bark containing functional phloem can be peeled off simply. For example, this strategy was used to investigate the distribution of BSV in highbush blueberry. A ring of the phloem-tissue-containing bark was removed from a section in a highbush blueberry stem, exposing the xylem-containing wooden part. Following this, BSV was inoculated below the girdling site. The appearance of the virus in the upper leaves of the host suggested that BSV can move independently of the phloem, likely by utilizing the xylem for translocation [14]. As an alternative to the mechanical girdling assay, a jet of steam can be used to induce cell death in the phloem of herbaceous hosts in which the mechanical separation of the phloem within the vasculature is challenging. The steam-treated assay has been applied in many herbaceous host–virus pathosystems. In one experiment, a portion of a cucumber stem was steam-treated, followed by inoculation with *Zucchini yellow mosaic virus* (ZYMV) at a site beneath the treated area. The detection of the virus in the xylem of the upper leaves demonstrated that ZYMV could enter xylem vessels and move [6]. Stem steam-treatments in other herbaceous host–virus pathosystems, which included *Turnip mosaic virus* (TuMV) in the *Nicotiana benthamiana* Domin [8], and *Southern bean mosaic virus* (SBMV) in the pinto bean (*Phaseolus vulgaris* L.) [26,28], resulted in similar findings. It is worth noting that stem-girdling and stem-steam-treatment assays alone are not sufficient to conclude that the virus could translocate via the xylem. The pith cells inside the girdled or steamed stem or a phloem bundle that occurs internal to the xylem [38] could provide a possible pathway for virus movement. Thus, a reliable conclusion must be drawn by combing these assays with other techniques, such as immunohistochemical detection and microscopy observations.

*In situ* observations based on fluorescence confocal microscopy and TEM could serve as straightforward methods to confirm the presence of a plant virus in xylem-associated cells. The thickened secondary cell walls in the mature xylem cells, which contain abundant cellulose and lignin, make them easily stained and recognizable. Confocal microscopy was used to examine the distribution of TuMV in the xylem of *N. benthamiana* by observing the localization of a green fluorescence protein (GFP)-tagged 6K2, a membrane-associated protein of TuMV [8]. Similarly, CTV was detected in the xylem of an infected citrus tree by monitoring the expression of free GFP produced from a modified CTV genome [7]. Compared to confocal microscopy, TEM provides a higher magnification for the direct visualization of virus particles. Based on TEM observations, the presence of virions and/or virion aggregates of LNYV, *Artichoke mottle crinkle virus*, *Carnation yellow fleck virus* (CYFV), and *Cucumber green mottle mosaic virus* (CGMMV), was detected in the xylem-associated cells mounted in the ultrathin sections of tobacco (*N. glutinosa*), artichoke (*Cynara Cardunculus var. scolymus* (L.) Benth.), carnation (*Dianthus caryophyllus* L.), and cucumber, respectively [9,22,31]. Often, nanoparticle-sized labels, such as immunogold particles, have been used in TEM imaging to help to recognize virions at a low density and validate virion aggregates in xylem-associated cells [34]. In addition to TEM and confocal microscopy, *in situ* immunohistochemical microscopy is used as an option for general low magnification optical microscopy. Virus particles are typically labeled with antibodies raised against the virus coat protein (CP), which are further detected by a secondary antibody conjugated with a chromogenic substrate. Virus detection is usually coupled with tissue staining (i.e., 5-bromo-4-chloro-3-indolyl phosphate/nitro blue tetrazolium for the xylem). This approach allowed the detection of tobacco (*Nicotiana tabacum* L. *cv. Samsun*) xylem cells harboring *Prune dwarf virus* and cucumber xylem cells harboring CGMMV as well as ZYMV or *Cucumber mosaic virus* (CMV) in co-infection with ZYMV [6,21,34]. Nevertheless, due to the uneven distribution of plant viruses in the plant hosts, locating virus-associated structures based on random two-dimensional image may lead to misinterpretations. The development of an *in situ* three-dimensional analysis can overcome these limitations by integrating data collected from serial sections [39]. Yet, undoubtedly, the direct observation of viruses in xylem-associated cells significantly strengthens the claim that plant viruses can blaze trails in the xylem.

In addition to the techniques mentioned above, the dot-blot immunoassay, *in situ* hybridization assay, and fluorescent antibody technique have been also used in some applications [14,37]. Unfortunately, methods allowing to isolate xylem-specific cells from the vasculature, which would aid our knowledge of the xylem functions and responses during virus invasion, are still lacking. It is expected that, with the development of research on xylem biology, the techniques toolkit will expand and advance our understanding of the xylem–virus interplay.

## 4. Viral Complexes in the Xylem

With high magnification microscopy, such as TEM, the virus-induced structures in the xylem tissue and sap can be documented in much detail. Although microscopy observations only capture information from a specific moment in virus infection, by assembling these snapshots into a bigger picture, we can put together a blueprint for virus biology in the host xylem.

The most frequently detected forms of a plant virus in the xylem are intact virions and virion crystalline aggregates. In French and Elder’s study, the particles of many plant viruses, including *Squash mosaic virus*, CMV, *Cowpea chlorotic mottle virus* (CCMV), and ZYMV, were detected in the guttation fluid of infected cucumbers [13]. It is assumed that the virion is the most stabilized structure for the virus in the xylem sap, as the CP subunits protect the virus genome against ribonucleases released upon programmed cell death (PCD) during tracheary element development [34,40,41,42]. However, some virions found in the xylem guttation could not maintain their infectivity. For instance, the virions of CCMV and ZYMV in the cucumber xylem guttation fluid lost their ability to initiate further infection in other plants with the reason remaining unclear [13]. Regardless, the presence of the virions in the xylem sap suggests that the virus could utilize the transpiration stream for potential spread within a plant. 

Crystalline virion arrays are frequently observed in xylem-associated cells, including tracheary elements (vessels and tracheids) and xylem parenchyma. For instance, in addition to being present in the phloem, ground parenchyma, and epidermis, hexagonal crystalline bundles of CYFV virions were seen in the mature xylem tracheary elements of infected spray carnation plants [22]. Likewise, crystalline virion patches of *Rice yellow mottle virus* (RYMV) accumulated in the xylem parenchyma cells and the phloem sieve elements of rice (*Oryza sativa* L.) were observed [27]. Such observations demonstrate that some plant viruses can form multi-virion aggregates in xylem-associated cells.

Other virus-induced structures observed for a number of viruses in xylem-associated cells are membrane-associated viral replication complexes (VRCs) and viral inclusion bodies. In virus-infected *N. benthamiana* plants, TuMV replication complexes were observed in the mature xylem vessels, in addition to being present in the phloem sieve elements [8]. *Potato virus Y* (PVY)-induced cytoplasmic inclusions were found in the epidermis, mesophyll, phloem parenchyma, phloem companion cells, and also in the xylem tracheary elements of *Solanaceous* plants [25]. The invasion of *Soilborne wheat mosaic virus* (SBWMV) into the mature xylem vessels of red winter wheat (*T. aestivum* L. *cv. Vona*) resulted in the formation of virus-induced inclusion bodies containing virion particles [32]. 

In addition to the accumulation of virus particles and VRCs, other virus forms, such as viral genomic RNA molecules, possibly in association with viral or host proteins, could also exist in xylem tissues. However, they have not yet been reported. Thus, exploring the potential virus forms or structures and understanding how they mediate virus interaction with and multiplication and/or transport in the xylem is the next challenge.

## 5. Possible Strategies for Virus Introduction into the Xylem

The findings of plant virus-induced VRCs and virion aggregations in xylem-associated cells, along with virus particles in the xylem sap, indicate that plant viruses can colonize xylem cells to complete their replication cycle and, subsequently, spread to distal plant tissues. From this point of view, the xylem and phloem play similar roles. Considering that the reported xylem-invading viruses belong to different families, each of which is involved in unique interactions with the host, a suite of different models of virus–xylem interplay may be appropriate. 

### 5.1. Vector-Mediated Introduction

**Figure 1:**
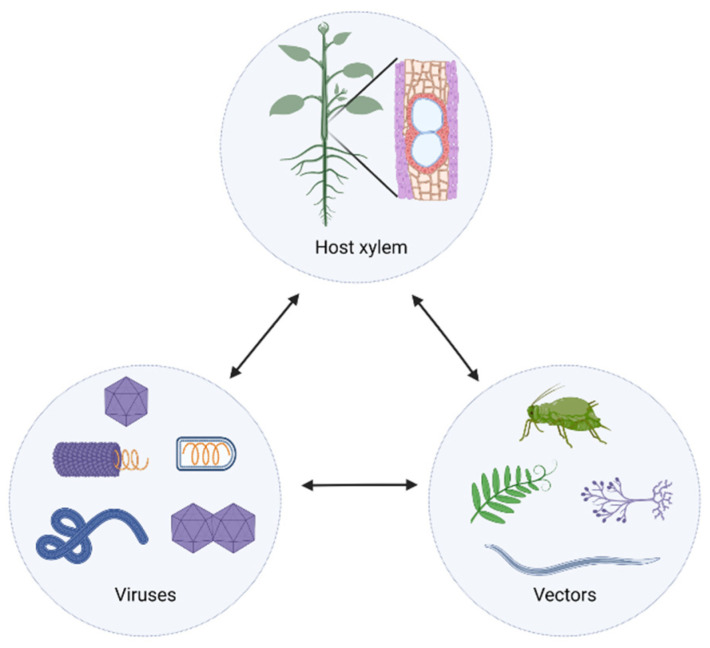
The interaction triangle depicting the interactions between viruses, vectors, and plant host xylem. The double arrowed lines stand for bidirectional interactions. The host xylem circle shows leaf, stem, and root xylem from both the herbaceous and perennial plants. The virus circle encompasses the xylem-invading viruses with different shapes and genome organization. The vector circle comprises the insects, fungi, parasitizing weeds, and nematodes. Figures were created with Biorender.com.

The presence of virus complexes in the xylem raises the question of how these viruses arrive to this destination. Under natural conditions, most plant viruses are introduced into a plant by vectors such as insects, nematodes, parasitic weeds, or fungi, with some viruses transmitted vertically through seeds. In the plant–vector–virus interaction triangle, vector-mediated transmission may directly introduce some viruses into the xylem (Figure 1). Aphids are the most common and efficient insect vector for the transmission of many viruses, including those that invade the xylem, such as PVY, BSV, and CTV. It is known that aphids occasionally consume the xylem sap to alleviate the osmotic effects of ingesting phloem juices, the concentration of which significantly exceeds that in xylem vessels [43]. In support of this statement, Saheed et al. observed that both the wheat xylem and phloem sustain severe damage due to Russian wheat aphid [44]. It is possible that the occasional feeding of aphids on xylem sap may initiate virus colonization in xylem-associated cells via direct contact. A similar mechanism may also be proposed in regard to nematode- and parasitic weed-mediated virus introduction. Levin et al. suggested that the nematode-borne *Tobacco ringspot virus* (TRSV) might be transmitted into the xylem during nematode feeding [45]. Furthermore, in order to acquire water and carbohydrates, the penetrating hyphae of dodder (*Cuscuta* sp.) penetrates into the host xylem and phloem and, thus, could form a path for virus introduction into the xylem, such as in the case described with CMV [46]. Regarding some viruses vectored by fungi, such as *Potato mop-top virus* transmitted by *Spongospora subterranean* (Wallroth) Lagerheim, BNYVV transmitted by *Polymyxa betae* Keskin, and CNV transmitted via *Olpidium bornovanus* (Sahtiyanci) Karling, it has been assumed that they are introduced through the fungus-penetrating hyphae generated during host colonization. Based on the anatomic examination of the *P. betae*-mediated BNYVV infection in sugar beet, Giunchedi and Pollini suggested that BNYVV reaches the xylem tissue of the taproot via a growing rootlet where the zoospores of the fungus have penetrated [19]. Such direct introduction of plant viruses into the xylem is rarely observed. However, a model depicting this possibility should not be ignored. 

### 5.2. Virus Movement through Plant Cell Connections

**Figure 2:**
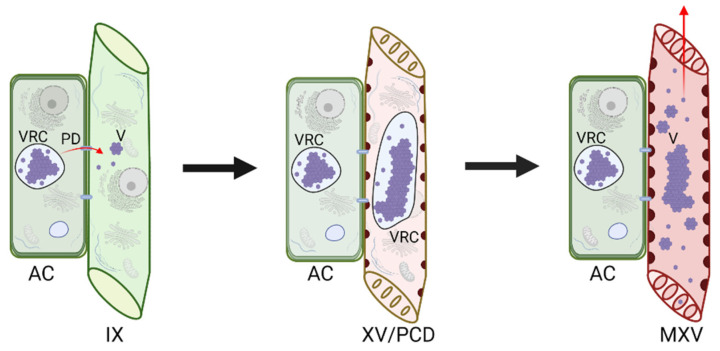
A model depicts how viruses enter immature xylem cells via cell-to-cell transfer from an adjacent cell. This model consists of three main stages: (1) Viruses enter the immature xylem cells, which still have the cellular machinery, via the plasmodesmata; (2) Viruses complete the replication and assembly cycle before the immature xylem cells undergo programmed cell death; (3) As the xylem vessels mature and the cell wall thickens, the cellular contents are released, and with that, the viruses are released into the xylem translocation stream for further spread. The red arrows point to potential virus movement routes. PD: plasmodesmata; VRC: viral replication complex; V: virus arrays; AC: adjacent cell; IX: immature xylem cells; XV/PCD: xylem vessels undergoing programmed cell death; MXV: matured xylem vessels. Figure was generated with Biorender.com.

**Figure 3:**
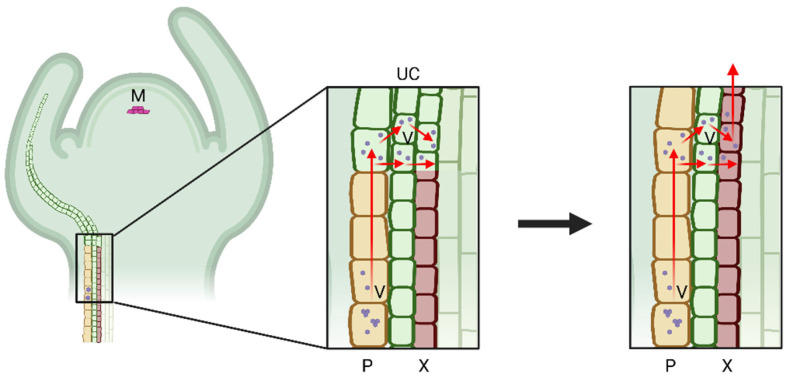
A virus–xylem invasion model illustrates a linkage between virus phloem transportation and virus cell-to-cell movement. The virus moves through the phloem to the distal part of the plant and infects all the undifferentiated cells beneath the meristem, including xylem precursor cells. With the maturation of the xylem cells, which undergo programmed cell death, the virus enters the xylem transpiration stream. The red arrows indicate the virus translocation route. M: meristematic tissue; UC: undifferentiated cells; V: virus; P: phloem; X: xylem. Figure was created with Biorender.com.

Usually, the xylem is not the only tissue that the virus colonizes. Thus, it is generally accepted that the xylem colonization by a plant virus results from niche expansion. The virus cell-to-cell movement-based model suggests that the xylem-residing viruses move there from adjacent non-xylem cells. It is well known that plant viruses can pass through modified plasmodesmata in a form of ribonucleoprotein complexes (RNP) or virions [47,48,49]. The viral entities move cell-to-cell between the adjacent cells of the epidermal, mesophyll, and phloem tissues until they reach the immature xylem cells. At this developmental stage, the immature xylem cells, which arise from the division of the cambial cells, have a full set of cytoplasm and cell organelles, allowing the virus replication cycle to proceed (Figure 2). This hypothesis is strongly supported by the time-lapse observation of the development of the xylem invasion by CTV. It was shown that, during the early stages of infection, CTV was mainly localized in the protophloem and metaphloem. With the infection continuing, CTV was able to translocate from the phloem-associated cells into the immature xylem cells, likely via the medullary ray, which is composed by a set of parenchyma cells that link the phloem and xylem [7]. As the xylem vessels mature by undergoing PCD, the viruses are released into the xylem translocation stream for further spread. Another model interlinks both the efficient long-distance virus transportation in the phloem conduit and a follow-up cell-to-cell movement towards the immature xylem. It proposes that, in order to enter the xylem, plant viruses first need to move longitudinally to distal parts of the host via the phloem conduit. Following that, the viruses exit the phloem sieve tubes via plasmodesmata and gradually infect all types of cells beneath the meristematic region, including the undifferentiated xylem cells. With the degeneration of the cellular contents, the xylem vessels mature and integrate with the xylem conduit by which the virus could achieve the xylem entrance (Figure 3) [13]. Both proposed models suggest that plant viruses can invade only the developing xylem and spread passively with the PCD-mediated maturation of the xylem cells. With that, virion crystalline arrays and VRCs remain inside the hollow xylem vessels, while the individual particles could enter the transpiration stream and eventually emerge in guttation [8,27,32]. Those two models explain how viruses successfully circumvent the membrane barriers during transport from the symplastic to apoplastic compartments.

**Figure 4:**
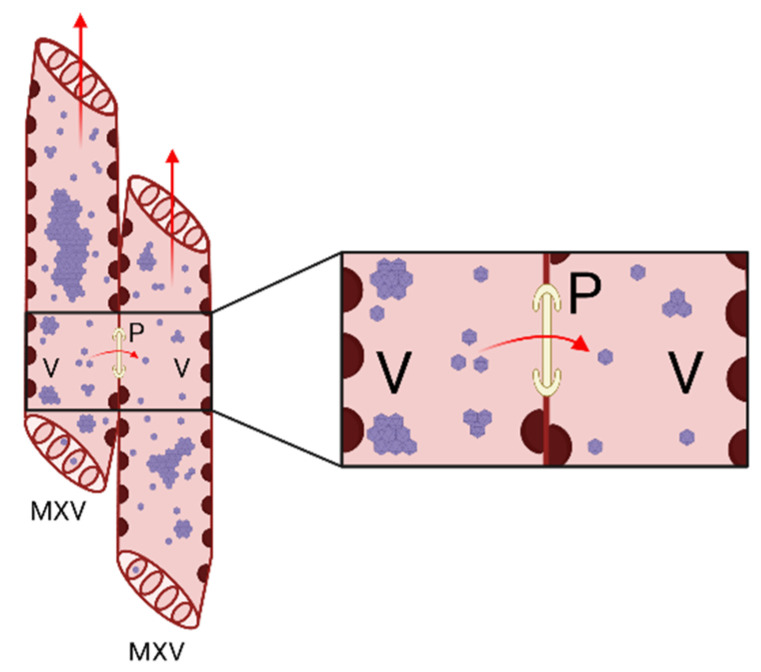
A model shows virus moment between two adjacent mature xylem cells. The mature xylem cells are connected by porous pits, which viruses can potentially use to move through. The red arrows indicate the virus translocation route. MXV: matured xylem vessel; V: virus; P: pit. Figures was created with Biorender.com.

We cannot exclude the possibility that the virions or RNPs of some viruses could move directly into mature xylem cells by some mechanisms because the movement of the virus through the thickened xylem cell walls is theoretically possible (Figure 4). Pits in the walls between xylem vessels would not be a strict barrier because the estimated pit membrane pore size could extend up to 840 nm in plants, which is larger than the diameter of most virus particles [50]. The particles of several viruses have been found in the pit membrane pores. It has been suggested that RYMV particles are transported to tracheary elements passively during the differentiation of parenchyma cells to vascular element cells that undergo PCD. However, the localization of RYMV virions over the vessel pit membrane pores suggests a pathway for the virus to migrate between vessels [27]. SBWMV was also proposed to enter juvenile xylem elements before PCD occurs and then spread upward with the maturation of the hollow vessels. Antibody-conjugated gold particles, which were used to label virions, were often observed in the pit pores between adjacent xylem vessels, suggesting that SBWMV may move laterally between adjacent xylem vessels [32]. There is also a possibility that the xylem-residing viruses could be unloaded from the mature xylem to infect other cells. This translocation requires the viruses to move through the thickened xylem cell wall. However, our understanding of the xylem unloading pathway for viruses remains incomplete and requires further investigation.

## 6. Factors Affecting Virus Xylem Colonization

Many host and viral factors, including proteins and even RNA motifs involved in virus phloem entry and transportation, have been identified [51]. Yet, the factors that influence virus xylem invasion are far from being known. It could be assumed that, as with those affecting virus phloem colonization, many virus and host factors could influence virus–xylem interactions. Here, we emphasize some of the identified factors from the perspectives of viruses, plant hosts, and vectors.

### 6.1. Viral Factors Influencing Xylem Invasion

The ability of plant viruses to enter the xylem tissue does not appear to correlate with the morphology of virus particles or their size, as the shapes of the virions as well as their dimensions are quite diverse. On the other hand, similar to the viral factors that mediate virus movement in the phloem, one can expect that virus-encoded proteins, especially the movement proteins and CPs, play key roles in the virus translocation into and within the xylem. However, our knowledge of those is essentially lacking. Yet, we know that certain alterations on the virus side can affect its ability to enter the xylem. Thus, the wild-type CTV variant of the T36 strain remains primarily limited to the phloem-associated cells in *Citrus macrophylla*. In contrast, a virus mutant with the deletion of the p33 protein gene is able to invade the immature xylem cells, which consequently results in the enhancement of disease [7,52]. Furthermore, virus coinfection may either promote or hinder the xylem invasion of another virus. For instance, the entry of CMV into cucumber xylem could be facilitated by co-infection with ZYMV [6]. This could be explained by the fact that co-infection with another virus provides the primary virus with extra movement proteins that facilitate xylem translocation. In contrast, the invasion of a strain of *Bean common mosaic virus* (BCMV), named “*Bean black root virus* NL3”, into the xylem of bean (*Phaseolus vulgaris* L. ‘Bataaf’) stems was delayed upon co-infection with another BCMV strain NY15. The NL3 migration from the primary leaves to the stem was hampered in the dual infection possibly due to lower virus loading or a shifted virus distribution as compared to those upon the single-virus inoculations [23,24]. Additionally, virus-imposed modifications of the plant cell structure can also influence virus interaction with the xylem. To this end, Opalka et al. proposed that the removal of Ca^2+^ ions from pit membranes by the capsid shells of sobemoviruses results in the disruption of the pit membranes and facilitates virion transport between vessels [27]. 

### 6.2. The Effect of Host on Virus Xylem Invasion

Plant host factors also play important roles in virus xylem invasion. This would be expected since viruses rely on the host machinery to complete the infection cycle, including replication, virion assembly, and movement in the xylem. Undoubtedly, host immunity could affect the ability of plant viruses to build a niche in the xylem. It is well known that the xylem acts as a field of battle between the plant hosts and vascular wilt pathogens [53]. The recognition of the pathogen-associated molecular patterns (PAMP) and effectors of the vascular pathogens by the extracellular and intracellular receptors in the xylem cells could lead to host PAMP-triggered and effector-triggered immunity responses. The activation of these plant xylem responses is usually accompanied by regulated expression of certain plant immunity-associated genes in the immature xylem cells [53]. It is unclear if the xylem exhibits similar responses once the invasion of a virus is recognized, as fewer studies investigating xylem-specific transcriptional and proteomic analyses in response to the xylem virus invasion have been reported. This is partially due to the difficulties in isolating xylem-specific tissues. With that, based on a study with TMV in *Arabidopsis thaliana* (L.) Heynhold and *N. benthamiana*, the phloem appears to have stronger transcriptional and translational alterations than the surrounding tissues, including xylem [54]. 

The formation of gel xylem occlusions is a common defense phenomenon observed in the xylem vessels against vascular wilt pathogens. For instance, the timely deposition of occluding gums and gels secreted by xylem parenchyma cells and tyloses could effectively trap the spread of the filamentous fungus *V. dahliae* [55]. Similar host physiological responses were recorded in the TuMV-infected Indian mustard (*Brassica juncea* (L.) Czernohorsky) plants. The invasion of TuMV into the vasculature not only triggered phloem necrosis, but also induced xylem gel occlusions [56]. Thus, the induction of such xylem obstructions may also contribute to restricting the virus xylem invasion. 

From the perspective of defense signaling, host PCD and reactive oxygen species (ROS) signaling are of importance. The accumulation of PVY cytopathological inclusion bodies in the xylem of *Solanaceous* plants corelated with the intensity of the host hypersensitive response [25]. In the citrus-CTV pathosystem, the accumulation of ROS in the citrus vasculature is thought to prevent the virus from entering into the xylem. This was evidenced by the observation that a CTV variant lacking the p33 protein, which, in contrast to the wild-type CTV, triggered weaker ROS accumulation and exhibited niche expansion by invading the xylem [17]. These data support the idea that the host immune responses hinder virus xylem invasion. On the other hand, another study showed that the presence of BMV in barley guttation fluid was correlated with the localized cell death response, especially in the xylem tracheary elements. The small patches of damaged cells within and adjacent to the veins potentially helped to release the virus, which further moved with the transpiration stream [20]. From this point of view, it seems that some host immune responses in the xylem could assist the virus transportation. Despite these diverse findings, the role of host factors in virus colonization of the xylem remains to be determined as no xylem-specific protein involved in this process has been identified.

### 6.3. The Role of Insect Vectors 

Acquisition and transmission by vectors are central to the infection cycle of most plant pathogenic viruses. It has been suggested that xylem invasion by viruses is aligned with vector transmissibility. Gergerich and Scott injected several purified viruses into the Black Valentine bean (*Phaseolus vulgaris* L.) or monarch cowpea (*Vigna unguiculata* L.) stem sections below the steam-treated nodes. Interestingly, only the beetle-transmissible viruses, SBMV, *Bean pod mottle virus*, and *Cowpea severe mosaic virus*, but not *Sunn-hemp mosaic virus* or TRSV, could successfully establish infection in the non-wounded tissue above the steam-killed area. This showed that the virus xylem translocation is associated with transmissibility by leaf-feeding beetles [26]. From the perspective of virus evolution, vector compatibility could affect the presence of plant viruses in the xylem for some insect-borne viruses. Due to the limited data, the contribution of vectors in xylem occupation by viruses remains to be understood. A possible explanation is that the vector-transmissible viruses may differ from the non-vector-transmissible ones in some property of their virions that determines binding with the host component. 

## 7. Conclusions

As more viruses are found to be associated with the xylem, our understanding of the importance of these largely overlooked interactions increases. The colonization of host xylem tissue provides plant viruses with a new niche to establish a successful infection. In particular, xylem invasion provides the virus with alternative routes for systemic spread, but also promotes virus acquisition by corresponding vectors. To date, the majority of the plant viruses found in the xylem is from the group of viruses with +ssRNA genomes, with a few that possess a −ssRNA or a ssDNA genome. The viruses can be classified into many different virus families and exhibit a range of diverse characteristics such as various shapes and unique genome organizations. Similarly, the diversity of plant hosts extends from herbaceous to perennial woody plants. *En masse*, hijacking the xylem cells seems to be a widely adapted strategy for plant viruses to promote infection in a broad range of plant hosts.

Despite a number of discoveries, most of which are discussed in this review, our understanding of virus–xylem interactions at the molecular level is still in its infancy. This is primarily caused by the difficulties tracing the virus buried in the xylem or isolating xylem-specific cells. However, thanks to the pioneering investigations of the interactions between viruses and the xylem tissue, a suite of virus–xylem interplay models that could explain virus entry and transport in the xylem conduit has been developed. In addition, some virus- and host-derived processes and factors that influence the xylem invasion by viruses have been identified. Next, we have yet to understand whether viruses spread via the xylem tissue unidirectionally or bidirectionally and how the virus entities traverse the plant cell walls. From the perspective of agronomy and horticulture, the investigation of virus–xylem interactions could provide potential solutions to manage severe virus-induced disease syndromes. Take CTV as an example, the invasion of CTV into the xylem tissue of citrus has been associated with the induction of stem pitting, a disease that is also commonly found in many tree crops, such as the apple crop infected with *Apple stem pitting virus*, grapevine infected with *Grapevine rupestris stem pitting-associated virus*, and stone fruit infected with *Tomato ringspot virus* [57,58,59,60]. Research focused on the interactions between viruses and the plant host xylem could aid in the understanding of the mechanisms of such diseases and the development of measures for their control.

## Figures and Tables

**Table 1:** Current list of viruses found in the xylem of herbaceous and perennial plant hosts.

	No.	Family	Genus	Species	Host Plants	References
+ssRNA	1	*Alphaflexiviridae*	*Potexvirus*	*Papaya mosaic virus*	Cucumber (*Cucumis surivus* L.)	[13]
	2	*Alphaflexiviridae*	*Potexvirus*	*Potato virus X*	*Nicotiana benthamiana* Domin	[8,18]
	3	*Benyviridae*	*Benyvirus*	*Beet necrotic yellow vein virus*	Sugar beet (*Beta vulgaris* L.)	[11,19]
	4	*Bromoviridae*	*Alfamovirus*	*Alfalfa mosaic virus*	Cucumber (*Cucumis surivus* L.)	[13]
	5	*Bromoviridae*	*Bromovirus*	*Brome mosaic virus*	Wheat (*Triticum aestivum* L.), Barley (*Hordeum vulgare* L.), Oat (*Arena sativa* L.)	[16,20]
	6	*Bromoviridae*	*Bromovirus*	*Cowpea chlorotic mottle virus*	Cucumber (*Cucumis surivus* L.)	[13]
	7	*Bromoviridae*	*Cucumovirus*	*Cucumber mosaic virus*	Cucumber (*Cucumis surivus* L.)	[6]
	8	*Bromoviridae*	*Ilarvirus*	*Prune dwarf virus*	Tobacco *(Nicotiana tabacum* L. *cv. Samsun*)	[21]
	9	*Closteroviridae*	*Closterovirus*	*Carnation yellow fleck virus*	Carnation (*Dianthus caryophyllus* L.)	[22]
	10	*Closteroviridae*	*Closterovirus*	*Citrus tristeza virus*	*Citrus macrophylla* Wester	[7]
	11	*Potyviridae*	*Potyvirus*	*Bean common mosaic virus*	Bean (*Phaseolus vulgaris* L. ‘Bataaf’)	[23,24]
	12	*Potyviridae*	*Potyvirus*	*Papaya ringspot virus*	Cucumber (*Cucumis surivus* L.)	[13]
	13	*Potyviridae*	*Potyvirus*	*Potato virus Y*	Potato (*Solanum tuberosum* L.) and tobacco (*Nicotiana tabacum* L.)	[25]
	14	*Potyviridae*	*Potyvirus*	*Turnip mosaic virus*	*Nicotiana benthamiana* Domin	[8]
	15	*Potyviridae*	*Potyvirus*	*Zucchini yellow mosaic virus*	Cucumber (*Cucumis surivus* L.)	[6,13]
	16	*Secoviridae*	*Comovirus*	*Bean pod mottle virus*	Black Valentine bean (*Phaseolus vulgaris* L.)	[26]
	17	*Secoviridae*	*Comovirus*	*Cowpea severe mosaic virus*	Monarch cowpea (*Vigna unguiculata* L.)	[26]
	18	*Secoviridae*	*Comovirus*	*Squash mosaic virus*	Cucumber (*Cucumis surivus* L.)	[13]
	19	*Secoviridae*	*Nepovirus*	*Tobacco ringspot virus*	Cucumber (*Cucumis surivus* L.)	[13]
	20	*Secoviridae*	*Nepovirus*	*Tomato ringspot virus*	Cucumber (*Cucumis surivus* L.)	[13]
	21	*Solemoviridae*	*Sobemovirus*	*Blueberry shoestring virus*	Highbush blueberry (*Vaccinium corymbosum* L.)	[14]
	22	*Solemoviridae*	*Sobemovirus*	*Rice yellow mottle virus*	Rice (*Oryza sativa* L.)	[27]
	23	*Solemoviridae*	*Sobemovirus*	*Southern bean mosaic virus*	Black Valentine bean, Pinto bean (*Phaseolus vulgaris* L.)	[26,28]
	24	*Tombusviridae*	*Gammacarmovirus*	*Melon necrotic spot virus*	Cucumber (*Cucumis surivus* L.)	[13]
	25	*Tombusviridae*	*Tombusvirus*	*Cucumber necrosis virus*	Cucumber (*Cucumis surivus* L.)	[13]
	26	*Tombusviridae*	*Tombusvirus*	*Tomato bushy stunt virus*	*Nicotiana benthamiana* Domin, *Gomphrena globose* L.	[29,30]
	27	*Tombusviridae*	*Tombusvirus*	*Artichoke mottle crinkle virus*	Artichoke (*Cynara Cardunculus var. scolymus* (L.) Benth.)	[31]
	28	*Virgaviridae*	*Furovirus*	*Soilborne wheat mosaic virus*	Red winter wheat (*Triticum aestivum* L. *cv. Vona*)	[32]
	29	*Virgaviridae*	*Pomovirus*	*Potato mop-top virus*	Tobacco (*Nicotiana tabacum* L. cv. Xanthi-nc)	[33]
	30	*Virgaviridae*	*Tobamovirus*	*Cucumber green mottle mosaic virus*	Cucumber (*Cucumis surivus* L.)	[13,34]
	31	*Virgaviridae*	*Tobamovirus*	*Maracuja mosaic virus*	*Nicotiana benthamiana* Domin	[35]
	32	*Virgaviridae*	*Tobamovirus*	*Pepper mild mottle virus*	Green pepper (*Capsicum annuum* L.)	[12]
	33	*Virgaviridae*	*Tobamovirus*	*Tobacco mosaic virus*	Tomato (*Solanum lycopersicum* L.)	[10]
	34	*Virgaviridae*	*Tobravirus*	*Tobacco rattle virus*	Potato (*Solanum tuberosum* L. *cv.* *Glada*), tobacco (*Nicotiana tabacum* L. *cv.* *Samsun*)	[36]
	35	*Virgaviridae*	*Tobamovirus*	*Tomato mosaic virus*	Tomato (*Lycopersicon esculentum* Mill.)	[12]
−ssRNA	36	*Rhabdoviridae*	*Cytorhabdovirus*	*Lettuce necrotic yellows virus*	Tobacco (*Nicotiana glutinosa* L.)	[9]
ssDNA	37	*Geminiviridae*	*Begomovirus*	*Tomato (yellow) leaf curl virus*	*Nicotiana benthamiana* Domin	[37]
	38	*Geminiviridae*	*Begomovirus*	*Tomato yellow leaf curl Sardinia virus*	*Nicotiana benthamiana* Domin	[37]
	39	*Geminiviridae*	*Begomovirus*	*Tomato golden mosaic virus*	*Nicotiana benthamiana* Domin	[37]

**Table 2:** Characteristic features of xylem-invading viruses.

Genome Composition	+ssRNA	+ssRNA	+ssRNA	−ssRNA	ssDNA
Family	*Bromoviridae*	*Benyviridae*	*Alphaflexiviridae*	*Rhabdoviridae*	*Geminiviridae*
	*Tombusviridae*	*Virgaviridae*	*Closteroviridae*		
	*Solemoviridae*		*Potyviridae*		
	*Secoviridae*				
	*Tombusviridae*				
Virion shape	icosahedral	short rigid rod-like	long flexuous	bullet-like	twinned-icosahedral
Virion size	25–35 nm/Diameter	65–350 nm/Length, 11~20 nm/Diameter	500~2000 nm/Length, 12~13 nm/Diameter	227 nm/Length, 66 nm/Diameter	~30 nm/Length, 18~20 nm/Diameter
